# Bedside resuscitative thoracotomy in response to cardiopulmonary arrest due to fatal hemothorax: a case report

**DOI:** 10.1093/jscr/rjag187

**Published:** 2026-03-22

**Authors:** Hitoshi Dejima, Masamichi Shinohara, Kanta Yamamoto, Kai Kubota, Shinya Kohmaru, Takuya Kato, Hirobumi Okawa

**Affiliations:** Department of General Thoracic Surgery, Shin-Kuki General Hospital, 418-1 Kamihayami, Kuki-city, Saitama 346-8530, Japan; Department of General Thoracic Surgery, Shin-Kuki General Hospital, 418-1 Kamihayami, Kuki-city, Saitama 346-8530, Japan; Department of General Thoracic Surgery, Shin-Kuki General Hospital, 418-1 Kamihayami, Kuki-city, Saitama 346-8530, Japan; Department of General Thoracic Surgery, Shin-Kuki General Hospital, 418-1 Kamihayami, Kuki-city, Saitama 346-8530, Japan; Department of General Thoracic Surgery, Shin-Kuki General Hospital, 418-1 Kamihayami, Kuki-city, Saitama 346-8530, Japan; Department of Surgery, Shin-Kuki General Hospital, 418-1 Kamihayami, Kuki-city, Saitama 346-8530, Japan; Department of Intensive Care Medicine, Shin-Kuki General Hospital, 418-1 Kamihayami, Kuki-city, Saitama 346-8530, Japan

**Keywords:** resuscitative thoracotomy, anterior thoracotomy, fatal hemothorax, postoperative bleeding

## Abstract

Anterior thoracotomy is an established approach in thoracic surgery, but life-threatening emergencies often occur under limited resources. We report a man in his 70s who developed cardiopulmonary arrest from massive postoperative hemothorax after right lower lobectomy. Resuscitative thoracotomy was required at the bedside before transfer to the operating room. With only a scalpel and scissors available, an anterolateral thoracotomy through the fifth intercostal space was extended cephalad by transecting costal cartilages, creating a hemiclamshell exposure within 1 minute. Manual hilar compression and gauze packing allowed temporary control, followed by definitive hemostasis in the operating room. The patient recovered without neurological sequelae or other complications following the resuscitative operation. This case highlights that bedside resuscitative thoracotomy can be performed successfully using only basic instruments.

## Introduction

Anterior thoracotomy procedures are well-established in the field of general thoracic surgery. However, resources such as facilities, equipment, and personnel are often limited in emergency scenarios, including life-threatening situations. Such circumstances necessitate clinicians to decide on the operative procedure and respond in a flexible manner. Herein, we report a case in which bedside resuscitative thoracotomy was performed on a patient who developed cardiopulmonary arrest due to fatal hemothorax.

## Case report

During a regular medical checkup for a man in his 70s, a chest computed tomography scan revealed an abnormal shadow corresponding to an irregular nodule 15 mm in size in the lower lobe of his right lung. The nodule was strongly suspected to be lung carcinoma because of high uptake intensity on 18F-2-fluoro-2-deoxyglucose positron emission tomography.

**Figure 1:**
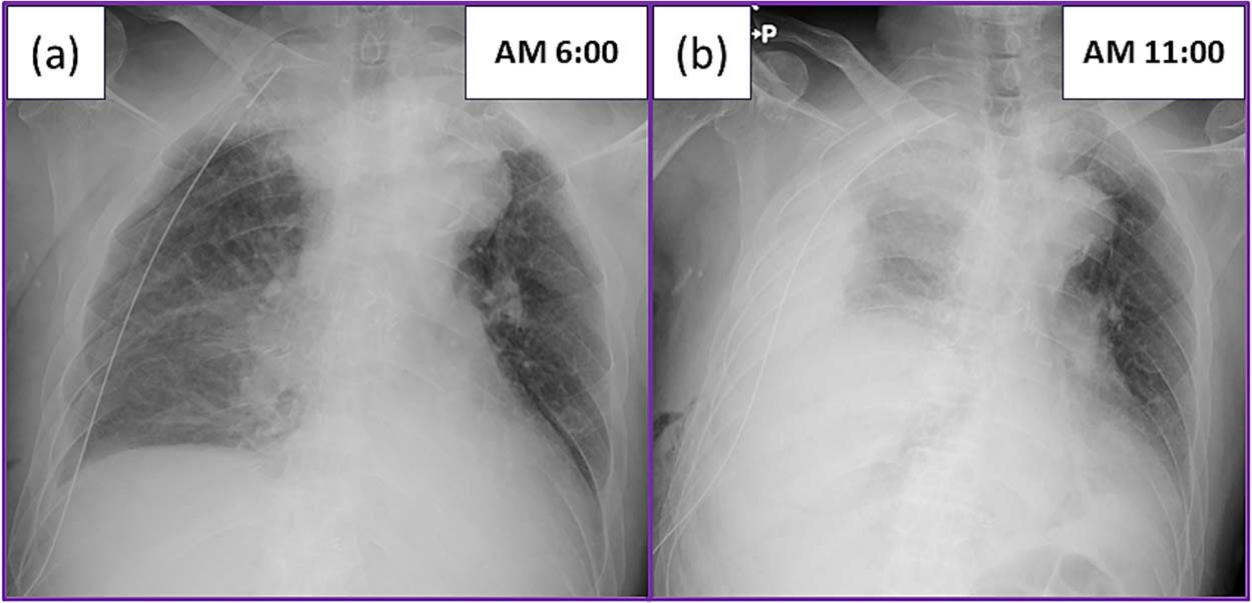
Postoperative chest X-ray: (a) Chest X-ray on the morning after surgery showed the expected postoperative changes. (b) The patient suddenly complained of dyspnea, and a large amount of bloody effusion drained from the chest tube after mobilization. The chest X-ray showed a large amount of effusion.

He underwent right lower lobectomy and mediastinal lymph nodes dissection, after non-small cell carcinoma was considered by intraoperative frozen section analysis during a partial resection. The operation was completed uneventfully in 170 minutes, with minimal blood loss (<10 mL). The postoperative course was also uneventful overnight, and blood tests and chest X-ray performed on the morning after surgery did not indicate any abnormalities (Fig. 1a). However, the patient suddenly complained of dyspnea, and a large amount of bloody effusion drained from the chest tube after mobilization. A chest X-ray confirmed the presence of a large amount of effusion (Fig. 1b). Suspecting massive bleeding, we clamped the chest tube and immediately started hyperhydration. However, while the operating room and blood transfusion were being prepared for reoperation, the patient experienced cardiopulmonary arrest following hemorrhagic shock. Bystander cardiopulmonary resuscitation, including tracheal intubation and chest compression, was immediately initiated by the intensive care unit medical staff. Because the operating room was not ready, we decided to perform resuscitative thoracotomy at bedside to apply direct digital pressure.

**Figure 2:**
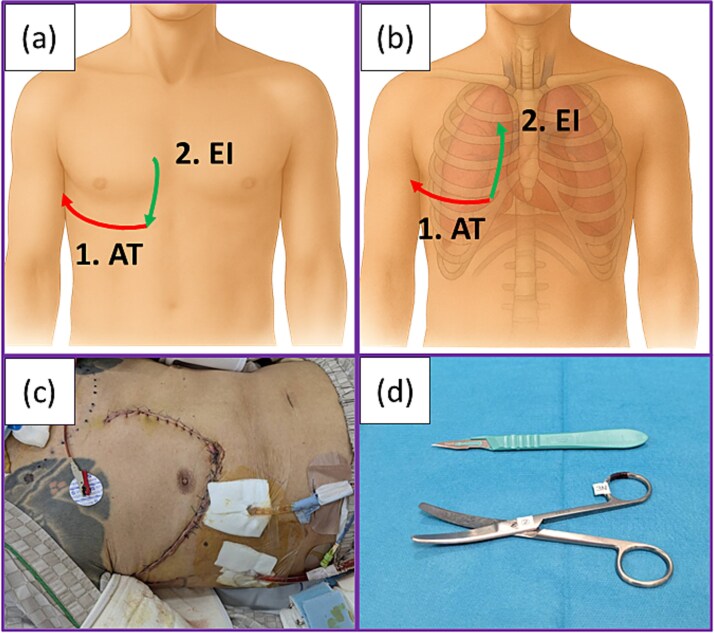
Procedure for resuscitative thoracotomy: (a) Skin incisions made in the present case. Anterolateral thoracotomy was first performed (1), followed by an extended incision directed caudally from the cephalad side according to the hand orientation with the scalpel (2). (b) Extended incision followed by transection of the cartilages was performed with scissors in a caudal-to-cranial direction (2). (c) Postoperative wound of the patient. (d) Only a scalpel and scissors were used to perform resuscitative thoracotomy in the present case. Abbreviations: AT: Anterolateral incision; EI: Extended incision.

An anterior incision was made along the fifth intercostal space using a disposable scalpel (Fig. 2a). The intercostal muscle was cut open using scissors to avoid lung injury, and thoracotomy was performed. The pulmonary hilum was blindly compressed manually using the left hand. Subsequently, the incision was extended cephalad along the right margin of the sternum, and the third to fifth costal cartilages were transected, thereby obtaining the surgical field that allowed identification of the pulmonary hilum (Fig. 2b). Gauze packing was performed around the pulmonary hilum, and manual compression was applied for hemostasis while the patient was transferred to the operating room. Although injuries were detected in the intercostal and internal mammary arteries, they were considered less significant because resuscitation was deemed to be the top priority. These injured arteries were ligated after thoracotomy. According to the medical record, this resuscitative thoracotomy approximately required 1 minute, and spontaneous circulation resumed during the procedure.

In the operating room, we explored the right thoracic cavity under general anesthesia. Bleeding was detected on the stump of the bronchial artery at the subtracheal bifurcation, which was dissected during mediastinal lymph node dissection. Hemostasis was achieved through artery ligation. Chest closure was achieved exclusively using surgical sutures, without incorporating prosthetic materials such as metal plates or synthetic meshes.

The postoperative course of the second operation was uneventful. No postoperative complications, including thoracic deformity or flail chest, were observed following resuscitative thoracotomy (Fig. 2c). The patient was extubated on postoperative Day 3, transferred to the rehabilitation section following acute care, and discharged on postoperative Day 62. The pathological diagnosis was combined small cell carcinoma with squamous cell carcinoma at Stage IIIA (pT2aN2M0). The patient independently visited the hospital 6 months after surgery for a regular medical checkup. No neurological sequelae after cardiopulmonary arrest due to hemorrhage were observed.

## Discussion

Several anterior thoracotomy procedures are well-established in the field of general thoracic surgery, including median sternotomy [1], clamshell incision [2], hemiclamshell approach [2, 3], trap-door thoracotomy [4, 5], anterior transcervical-thoracic approach (Dartevelle technique) [5, 6], transmanubrial approach [6], and anterolateral thoracotomy. Of these, clamshell thoracotomy, anterolateral thoracotomy, and median sternotomy have been reported to be widely used in the emergency setting. Clamshell thoracotomy, in particular, is more likely to be used on patients needing lung resection or cardiac repair. Compared with other approaches, this approach has a higher success rate because it allows for better organ repair without increasing systemic or thoracic complications [7–10].

However, emergency settings preclude adequate preparation. Resuscitative interventions are often performed under constraints of personnel, equipment, and facilities. In clamshell thoracotomy, division of the sternum is mandatory, and the standard instrument for this procedure is an oscillating sternal saw. Several alternatives have been described for when time is critical and specialized equipment are not immediately available, including heavy trauma shears, a Lebsche knife with mallet, or a Gigli saw [11, 12]. In the present case, cardiopulmonary arrest due to massive hemorrhage occurred on the bed of the intensive care unit. Resuscitative thoracotomy was required, but none of the aforementioned instruments were at hand. The only surgical equipment available at the bedside were a scalpel and scissors (Fig. 2d). The patient could not be positioned in the decubitus position during chest compression. In these circumstances, right anterolateral thoracotomy was the only feasible procedure. In this procedure, a skin incision is typically made along the inframammary fold in women and caudal to the areola in men, which generally corresponds to the fifth intercostal space. However, anterolateral thoracotomy involves a relatively small incision resulting in a limited surgical field, which may not be optimal for time-sensitive interventions [8]. Additional exposure may be useful for facilitating certain operative procedures [7]. Since the costal cartilages could be transected using scissors, they were divided cephalad along the right margin of the sternum, thereby exposing a surgical field similar to that obtained in hemiclamshell thoracotomy.

Postoperative massive bleeding is a rare but life-threatening adverse event for patients, and it often occurs under constrained circumstances. The procedure reported herein serves as an option for resuscitative thoracotomy.
